# Argon Humidification Exacerbates Antimicrobial and Anti-MRSA kINPen Plasma Activity

**DOI:** 10.3390/life13020257

**Published:** 2023-01-17

**Authors:** Ramona Clemen, Debora Singer, Henry Skowski, Sander Bekeschus

**Affiliations:** 1 ZIK Plasmatis, Leibniz Institute for Plasma Science and Technology (INP), Felix-Hausdorff-Str. 2, 17489 Greifswald, Germany; 2 Clinic and Policlinic for Dermatology and Venerology, Rostock University Medical Center, Strempelstr. 13, 18057 Rostock, Germany

**Keywords:** *Candida albicans*, *Escherichia coli*, gas plasma technology, kINPen, plasma medicine, *Pseudomonas aeruginosa*, *Pseudomonas* spp., reactive oxygen species, ROS, *Staphylococcus aureus*, *Staphylococcus epidermidis*

## Abstract

Gas plasma is a medical technology with antimicrobial properties. Its main mode of action is oxidative damage via reactive species production. The clinical efficacy of gas plasma-reduced bacterial burden has been shown to be hampered in some cases. Since the reactive species profile produced by gas plasma jets, such as the kINPen used in this study, are thought to determine antimicrobial efficacy, we screened an array of feed gas settings in different types of bacteria. Antimicrobial analysis was performed by single-cell analysis using flow cytometry. We identified humidified feed gas to mediate significantly greater toxicity compared to dry argon and many other gas plasma conditions. The results were confirmed by inhibition zone analysis on gas-plasma-treated microbial lawns grown on agar plates. Our results may have vital implications for clinical wound management and potentially enhance antimicrobial efficacy of medical gas plasma therapy in patient treatment.

## 1. Introduction

Microorganisms are the foundation of life by keeping homeostasis on a large scale in ecosystems and on a small scale in cooperating commensal and symbiotic behavior in animals and plants. However, some microorganisms or a lack of host organisms’ defense can lead to severe infection and compromised organ function, including in the skin [1,2]. For instance, if wounds become infected or the wound’s host is deprived of the ability to clear the infection, chronification and ulceration occur, leading to hampered wound healing and long-term reduction of quality of life [3,4]. There are many different approaches to clearing wound infection and supporting wound healing available on the market [5,6]. About ten years ago, one particular technology was approved for treating non-healing and infected wounds in Europe, cold physical plasma [7]. The leap innovation of this technology was the ability to generate partially ionized gases in ambient air operated at body temperature so that no thermal harm was provoked when treating cells and tissues. The treatment with cold physical plasma has been shown to reduce the number of microorganisms in wounds [8,9]. A recent randomized, controlled clinical trial [10] suggested that cell-stimulating mechanisms also promote wound closure besides the known antimicrobial activity of gas plasma technology [11]. However, it remains established that reactive oxygen species (ROS) are a major mechanism of gas plasma therapy in vivo [12].

Antimicrobial effects of cold (body-temperature) gas plasma devices were described in the mid-1990s for the first time [13], with historic plasma medicine applications dating back to the turn of the 19th to 20th century [14]. Today, it is known that the share of ultraviolet (UV) and VUV radiation in this effect is relatively negligible, at least for plasma jets [15,16]. Similar findings were made for the portion of electric fields to the effects observed. Many studies have revealed and summarized the antimicrobial effects of gas plasma-derived ROS [17,18]. This was found mainly by adding antioxidant molecules or enzymes, such as N-acetylcysteine and catalase, to the samples, finding that many of the gas plasma-derived effects were reversed. However, only a few have convincingly shown how to optimize antimicrobial effects of existing gas plasma devices [19,20]. With many device configurations, especially gas plasma jets, the resulting ROS mixture expelled depends on the feed gas composition. For instance, partially opposing chemical pathways can be forced by modulating the presence and quantity of nitrogen or oxygen in the immediate surrounding or feed of plasma jets [21,22].

It is known that the gas plasma jet kINPen is the only device certified for medical conditions worldwide [11]. The device is operated with argon gas feed gas. However, to date, no study has convincingly shown an antimicrobial efficacy optimization of this argon plasma jet. To this end, we tested several feed gas conditions and identified humidity as a promising approach with the potential to optimize current wound healing therapeutic strategies based on commercially available gas plasma technology. Nitrogen, oxygen, or humidified argon were added to dry argon gas as feed gas conditions, as nitrogen and oxygen provoke the increased generation of reactive nitrogen and oxygen species, respectively, while humidity was shown to increase the levels of hydroxyl radicals [23].

## 2. Materials and Methods

### 2.1. Culture of Microorganisms

**Figure 1:**
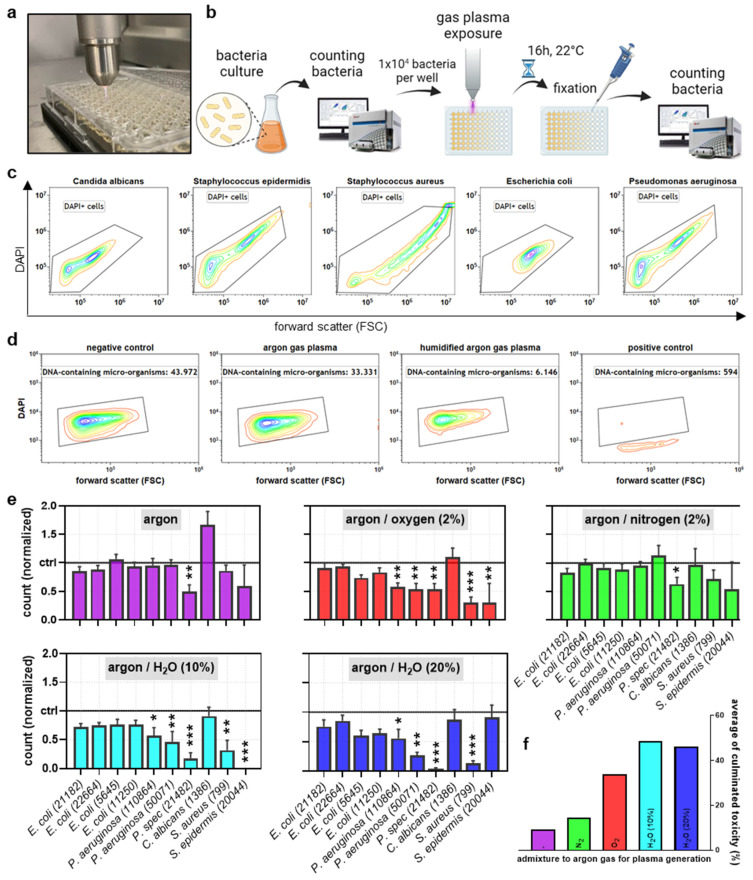
**Study design and plasma feed gas optimization.** (**a**) representative image gas plasma treatment procedure; (**b**) study overview; (**c**,**d**) representative flow cytometry dot plots of FSC vs. DAPI of different microorganisms (**c**) and *P. aeruginosa* (50071) that had remained untreated (negative control) or was exposed to dry (5 s) or humidified (5 s) argon gas plasma or positive control solution (**d**); (**e**) quantitative cell counts of the microorganisms used in this study 16 h post gas plasma exposure with different feed gas conditions; (**f**) cumulative sum-score of antimicrobial effect across all microorganisms tested for each feed gas condition. Data are representative (**c**,**d**) or mean + S.E. (**e**) of two experiments. Statistical analysis was performed using one-way analysis of variances with * = *p* < 0.05; ** = *p* < 0.01; and *** = *p* < 0.001.

Several types of microorganisms were used in this study, and all were received from the German Collection of Microorganisms and Cell Cultures (Leibniz Institute DSMZ, Braunschweig, Germany). Among them were bacteria, such as *Escherichia* (E.) *coli* (DSMZ reference numbers 21182, 22664, 5645, and 1125), *Pseudomonas* (P.) *aeruginosa* (DSMZ reference numbers 110864 and 50071) and species (DSMZ reference number 21482), *Staphylococcus* (S.) *aureus* (DSMZ reference number 799), and *S. epidermidis* (DSMZ reference number 20044), and yeast, such as *Candida* (C.) *albicans* (DSMZ reference number 1386). Microorganisms were cultured in broth as indicated suitable for each strain type, as DSMZ instructions recommended before gas plasma exposure (Figure 1a).

### 2.2. Feed Gas Alterations and Gas Plasma Treatment of Microorganisms

The atmospheric pressure argon plasma jet kINPen (neoplas, Greifswald, Germany) was utilized in this study. This device’s electrical and chemical characteristics have been described in detail before [24]. Its excitation frequency is approximately 1 MHz, and its nominal output power is about 1 W. It has been operated at one standard liter per minute of argon gas (purity: 4.6; Air Liquide, Bremen, Germany). For exposure of samples, such as microbial cultures placed in flat-bottom 96-well plates (Sarstedt, Sarstedt, Germany), the setup was as previously described [23]. Briefly, the gas plasma jet was installed (Figure 1a) on a computer-controlled xyz-stage (CNC step HIGH-Z edition). The distance of the jet to the treated liquid (0 mm, conductive mode [25]), the jet’s relative position to the well (always: centered), and the exposure time (see legends) were precisely controlled and monitored using appropriate CNC-compatible software. For the addition of oxygen (purity: 4.5; Air Liquide) and nitrogen (purity: 4.5; Air Liquide), a panel of mass flow controllers and valves was used and controlled through a central digital panel (MKS, München, Germany) for sub-percent precision. Similarly, humidified argon was generated by bubbling the gas through double-distilled water before being mixed with dry argon. For the treatment of microorganisms, the respective strains were seeded at 1 × 10^4^ microorganisms per well in 100 µL of each broth. Evaporation of liquid through gas plasma exposure was accounted for by adding a predetermined amount of sterile double-distilled water. Microorganisms were counted by taking 20 µL and adding paraformaldehyde before measuring via flow cytometry as described in Section 2.3. To control the growth of unspecific bacteria contamination, 100 µL of broth without bacteria was treated. In some experiments, H_2_O_2_ was added instead of gas plasma exposure. The rest of the experimental workflow and analysis remained the same.

### 2.3. Flow Cytometry

Following gas plasma exposure, the microorganisms in the microwell plates were kept at room temperature in the dark for 16 h. In some conditions, 2 µL of catalase solution (Sigma-Aldrich, Taufkirchen, Germany) was added (final concentration: 20 µg/mL) either before or after gas plasma exposure of samples. Then, to each well, fixative was added (4% paraformaldehyde; Sigma-Aldrich) and incubated for 10 min in the dark. Then, to each well, 4′,6-diamidino-2-phenylindole (DAPI, final concentration 10 µM; BioLegend, Amsterdam, The Netherlands) was added, and the microorganisms were incubated for 15 min in the dark. All microorganisms contain ample amounts of DNA to which DAPI binds and becomes fluorescent. This way, microorganisms can be conveniently detected using flow cytometry. Next, the microplates were added to an autosampler of a CytoFLEX S flow cytometer (Beckman-Coulter, Krefeld, Germany) trigger through the forward scatter (488 nm laser diode) and collection of DAPI fluorescence via λ_ex_ 405 nm and λ_em_ 450 ± 45 nm. After mixing by the autosampler, 100µL of cell suspension was acquired from each well. The resulting .fcs (3.1 standard) data files were analyzed using Kaluza analysis software 2.1.3 (Beckman-Coulter).

### 2.4. Antimicrobial Efficacy Using Agar Plates

Sixteen hours following gas plasma exposure, the resulting microorganism samples were spread onto CASO agar plates at different dilutions. The plates were incubated at 30 °C overnight. After 24 h, colony formation was quantified by manual counting, whereas the threshold of countability was set to 1000 per plate. For measuring the inhibition zone, bacteria were plated on agar plates, and the middle was treated with plasma. The plates were incubated at 30 °C overnight, and the area was measured after 24 h by inhibition zone image analysis.

### 2.5. ROS Analysis

Phosphate-buffered saline (PBS; Pan-Biotech, Aidenbach, Germany) was exposed to gas plasma for different treatment times. A gradient of humidified feed gas was applied to a set of samples. Subsequently, hydrogen peroxide (H_2_O_2_) was quantified using the Amplex Ultra Red Assay Kit (Thermo Fisher Scientific, Dreieich, Germany) as previously described [23]. In addition, nitrite (NO_2_^−^) concentrations were assessed using Griess reagent (Thermo Fisher Scientific) as recently outlined [26].

### 2.6. Statistical Analysis

Statistical analysis was performed using prism 9.5.0 (GraphPad Software, San Diego, CA, USA) based on one-way analysis of variances as indicated in the figure legends. Data are shown as mean + S.E. if not described differently, and the number of experiments is given in the legends. The alpha error was set as follows: α = 0.05 (*), α = 0.01 (**), and α = 0.001 (***).

## 3. Results

### 3.1. Comparison of Plasma Jet Feed Gas Admixtures for Abolishing Microbial Growth

This study sought to investigate the antimicrobial efficacy of the atmospheric pressure argon plasma jet kINPen operated at different feed gas conditions to identify those potentially increasing the current capacity targeting microorganisms. To this end, a diverse set of microorganisms was exposed to gas plasma in vitro, and microbial growth was analyzed 16 h later (Figure 1b). While these different strains and organisms gave different patterns in size and nucleic acid content, all could be quantified confidently (Figure 1c). Hence, toxicity to microorganisms inflected by gas plasma treatment or positive control (10% ethanol) could be quantified properly (Figure 1d). Next, we screened 10 different microorganisms (9 bacteria, 1 yeast) for increased sensitivity to argon gas plasma exposure (treatment time per well: 5 s) admixed with either oxygen, nitrogen, or humidified argon (Figure 1e). *E. coli* strains were less sensitive, while the yeast (*C. albicans*) was resistant to the gas plasma treatment. By contrast, *P.* strains showed a particular sensitivity, which was partially also the case for *S.* strains. Concerning the gas plasma treatment regimens, a reduction of absolute cell numbers was observed for all regimens investigated, while admixtures were more potent than dry argon gas alone. This was also reflected in the cumulative microbial reduction scores for each feed gas regimen across all ten strains investigated (Figure 1f), which was the highest for humidified argon gas plasma treatment.

### 3.2. Comparison of Plasma Jet Feed Gas Admixtures for Inhibition Zones and Abolishing CFU

**Figure 2:**
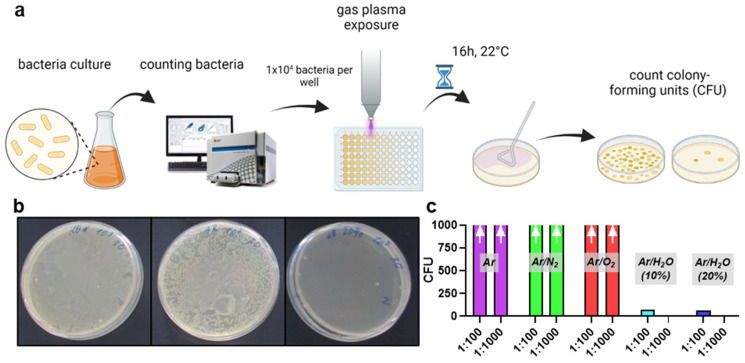
**Colony formation assay confirmation of plasma feed gas optimization.** (**a**) assay design; (**b**) representative agar plates with colony-forming units (CFU) of untreated (**left**), 30 s dry argon-plasma-treated (**middle**), and 30 s humidified argon-plasma-treated cell suspensions of *E. coli*; (**c**) CFU quantification of *E. coli* exposed to gas plasma of different feed gas conditions; arrows indicated a too-high number of CFU for manual counting. Data are representative of three experiments.

**Figure 3:**
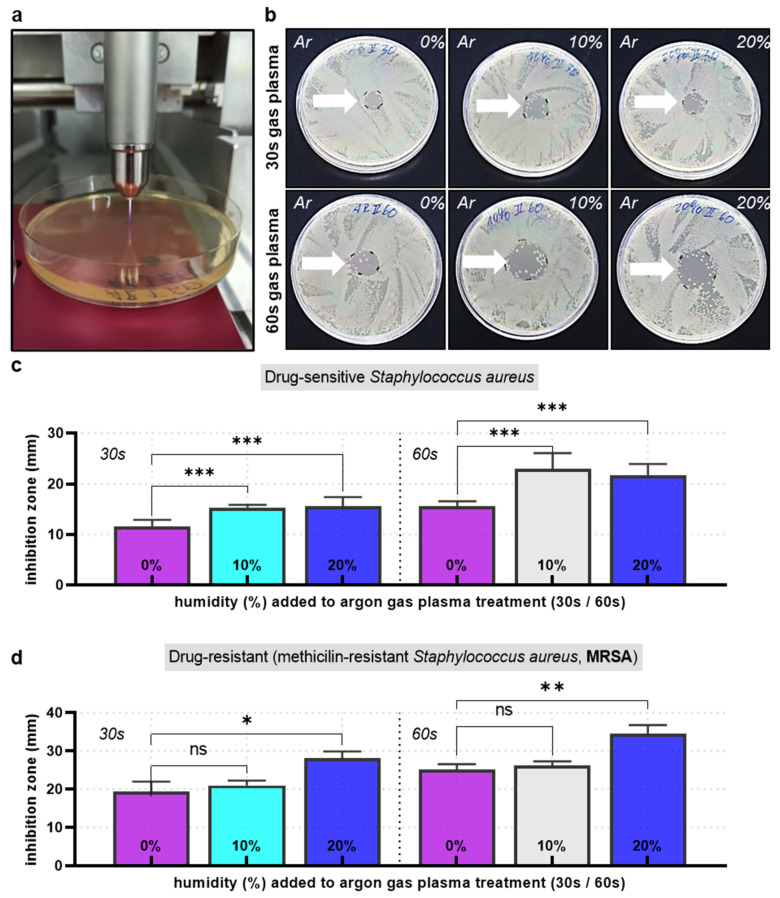
**Inhibition zone analysis.** (**a**) image of the kINPen treating *S. aureus* plated on agar plates; (**b**) representative agar plates with gas-plasma-generated (treatment times: 30 s or 60 s) inhibition zones for dry and humidified argon feed gas conditions; (**c**,**d**) inhibition zone quantification in drug-sensitive (**c**) and drug-resistant (MRSA, (**d**) bacteria; data are representative (**b**) or mean + S.E. of three experiments; statistical analysis was performed using one-way analysis of variances with * = *p* < 0.05; ** = *p* < 0.01; and *** = *p* < 0.001.

To confirm the results retrieved by flow cytometry of microorganisms grown in suspension, we plated the gas-plasma-treated *E. coli* samples on agar plates. We investigated the number of colony-forming units (CFU) per sample and at different dilutions (Figure 2a). Macroscopically, the efficacy of humidified argon-gas-plasma-treated samples in reducing CFUs (Figure 2b, right image) was apparent to be superior to that of dry argon-gas-plasma-treated microorganisms (Figure 2b, center image) and untreated controls (Figure 2b, left image). Quantitative CFU assessment strengthened this notion, concluding a superior ability of humidified argon gas plasma to decelerate microbial colony formation (Figure 2c). To further confirm the superiority of humidified over dry argon gas plasma treatment of microorganisms, *S. aureus* was plated on agar plates, and the centers of the plates were exposed to gas plasma for 30 s or 60 s (Figure 3a). The plates were incubated overnight and photographed (Figure 3b), followed by quantifying inhibition zones (Figure 3c). Expectedly, 30 s gas plasmas treatment created smaller inhibition zones compared to 60 s. Interestingly, and similar to the previous experiments in this study, humidified argon gas plasma was significantly superior regarding inhibition zones when compared to dry conditions. It should be noted, however, that 20% argon feed gas humidity was similar or slightly less effective compared to 10% humidity, as already observed in the microorganism suspension gas plasma experiments (Figure 1f). Strikingly, a significantly increased inhibition zone was also observed in drug-resistant *S. aureus* (MRSA) (Figure 3d).

### 3.3. ROS and RNS Analysis

**Figure 4:**
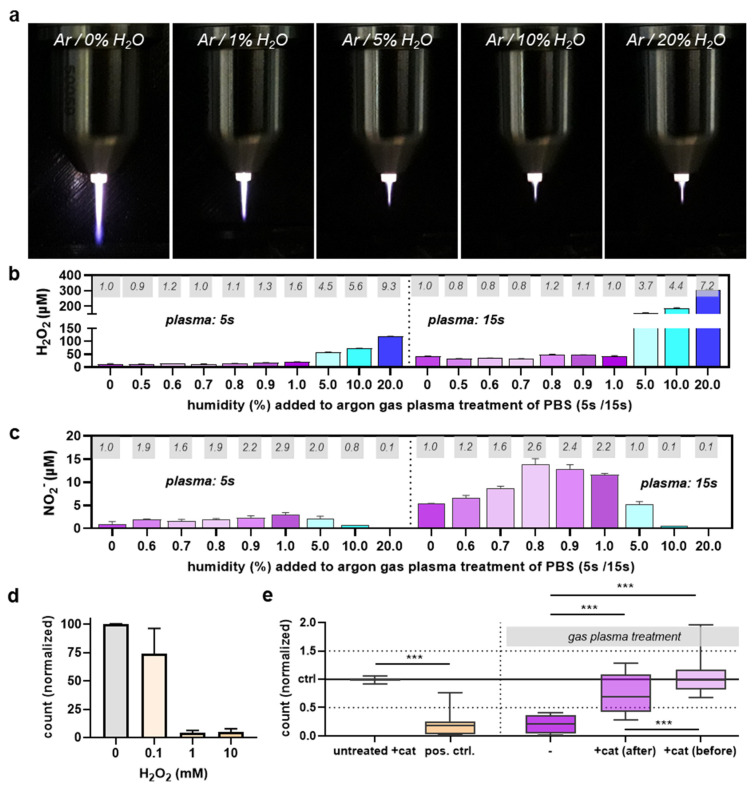
**ROS dependence on argon feed gas humidity and antimicrobial role of H_2_O_2_.** (**a**) macroscopic images of the kINPen argon plasma jet operated at different humidity admixtures; (**b**,**c**) H_2_O_2_ (**b**) and NO_2_^−^ (**c**) levels in dependence of feed gas humidity of argon plasma with grey boxes indicating fold-change of concentration to dry (0% humidity) argon gas plasma treatment; (**d**) H_2_O_2_ dilution series and antimicrobial effect in *P. aeruginosa* 16 h later as assessed using flow cytometry; (**e**) flow cytometry microbial growth analysis 16 h after exposure to positive control (ethanol), dry gas plasma (−), dry gas plasma treatment followed by catalase addition (+cat (after)) or following catalase addition (+cat (before); data are representative (**a**–**d**) or boxplots showing median of four experiments with *** = *p* < 0.001.

The major mode of action in gas plasma is ROS generation. Identifying alterations in ROS generation signatures can reveal what types of ROS potentially contribute to the effects observed. To this end, we used different feed gas humidity percentages and exposure of liquid to better understand ROS dynamics. Macroscopically, it could be observed that already 1% humidity in the argon feed gas led to a shorter kINPen plasma effluent (Figure 4a). Increasing humidity to 5% increased this effect, while the length between 10% and 20% humidity was unchanged, indicating a limit of this shortening effect. Subsequently, H_2_O_2_ was quantified in PBS exposed to 5 s or 15 s of dry or humidified argon gas plasma. As expected from previous reports, there was an increased H_2_O_2_ generation with highly humid argon plasma kINPen treatments (Figure 4b). However, three novel observations were made. First, it was interesting to note that the H_2_O_2_ production rates did not change at humidity percentages up to 1%, despite an apparent visual change of the plasma jet plume at that percentage (Figure 4a). Second, it has not been described that adding feed gas humidity elevates H_2_O_2_ generation rates more than 9-fold. Third, we found that during humidity concentrations of up to 1%, nitrogen species such as NO_2_^−^ were elevated (Figure 4c). Their concentrations declined at 5% or higher, being close to the detection range at 20% humidity. As we could not detect any enhanced antimicrobial activity of feed gas humidity up to 1% (data not shown), while marked elevation was observed at 10% and 20%, where H_2_O_2_ levels were also found to be highest, we investigated its role in the effects observed. As proof of principle, we tested a dilution series of H_2_O_2_ for its antimicrobial activity in *E. coli*, and the expected decline was found (Figure 4d). Next, we did the reverse experiment by using catalase, a potent scavenger of H_2_O_2_ [27], which was added either before or immediately after gas plasma exposure of four different microorganism strains. The summarized data indicate that catalase added prior to gas plasma treatment of microorganisms in liquid suspensions (not on agar plates) completely and significantly abrogated antimicrobial gas plasma effects (Figure 4e). Interestingly, catalase addition after gas plasma exposure only partially rescued the demise of microorganisms. These data strongly suggested that H_2_O_2_ plays a decisive role in the enhanced antimicrobial activity of humidified argon plasma jet treatments.

## 4. Discussion

Our study aimed at identifying feed gas compositions that enhanced the antimicrobial activity of the clinically employed atmospheric pressure argon plasma jet kINPen. Here, we report highly humidified argon gas feed into the plasma jet to meet this aim. We found elevated antimicrobial activity across several assays.

The idea of feed gas alterations in gas plasma devices to enhance or reduce a given effect in plasma medicine has been around for over a decade [28]. The three larger fields addressed with such an approach so far are wound healing [11], oncology [29], and decontamination and antimicrobial activity [17,30]. Regarding the latter and focusing on the kINPen, a previous version of this atmospheric pressure argon plasma jet (kINPen 09) operated with either dry argon or dry argon plus 1% oxygen did not yield better results concerning antimicrobial activity [31]. In contrast, small oxygen admixtures (<1%) have been previously reported to increase inhibition zones in argon-gas-plasma-treated *S. aureus* cells plated on agar plates [20]. Notwithstanding, it should be noted that due to biofilm formation and high abundance of ROS-scavenging biomolecules in tissues, the absolute antimicrobial activity (in terms of log CFU reduction) is only modest in gas-plasma-treated wounds [8,9,11] compared to ideal laboratory conditions where several log reduction of CFU are often observed [32].

It is known that the chemistry of gas plasma sources depends on the feed gas and surrounding gas. The same is true for the kINPen [21,33,34]. This includes feed gas humidification, which has been described to lead to enhanced generation of H_2_O_2_ in kINPen-treated liquids [23,35]. Such humidification leads to a change in the species composition in the kINPen effluent, including a decline of atomic oxygen, nitrogen species, ozone, and superoxide radicals and an increase of hydroxy [23,36]. Importantly, when referring to humidity, it is referenced to feed gas humidity, as the impact of environmental (ambient air/room air) humidity on the species profiles generated is negligible [36]. However, our finding that small humidity admixtures of around 0.8% added to the feed gas promote the generation of reactive nitrogen species, of which NO_2_^−^ is a reaction product, has not been documented before. Future studies may underline the mechanisms of these findings by performing additional analysis of the plasma jet via, e.g., optical emission spectroscopy. This information could be valuable for the field of plasma agriculture, where nitrogen fixation into liquids is one primary goal [37]. If in situ generation of H_2_O_2_ is aimed to be maximized, such as in the application of plasma-treated or plasma-condition media (also referred to as PAM or PAL) [38], high feed gas humidification rates may help achieve this goal, depending on the plasma source used.

Since H_2_O_2_ is a central molecule in redox biology and signaling [39], enhanced kINPen treatment effects on HaCaT keratinocytes were identified using humidified over dry argon gas [35]. Such a setting was also more cytotoxic in B16F10 melanoma cells in vitro, while nitrogen and oxygen admixtures were less effective compared to dry argon gas kINPen operation [23]. Therefore, our results are in line with previous findings made in eukaryotic cells with regard to humidified argon kINPen operations. However, in our current study, the additive toxicity conferred by such humidification in the eukaryotic organism *C. albicans* was rather modest, albeit previous findings had shown an antifungal activity of the kINPen in the agar plate model [40]. This difference might be explained by the different exposure models in our current study, foreseeing antimicrobial gas plasma treatment of cells suspended in broth rather than plated on agar. In addition, it must be noted that our results need further investigation in other types of plasma sources and, as of now, holds for the kINPen, primarily. We have recently humidified the helium feed gas of the European reference jet (also referred to as COST jet) [41], and our results did not show an enhanced H_2_O_2_ production or toxicity in eukaryotic cells exposed to this humidified feed gas compared to dry helium gas plasma conditions [42].

In summary, our results on the enhanced antimicrobial activity of humidified argon plasma appear promising for wound decontamination purposes. It must be tested in future animal and patient studies to verify its potential.

## Data Availability

The data can be retrieved from the corresponding author upon reasonable request.
